# Evaluation of the detection accuracy of set-up for various treatment sites using surface-guided radiotherapy system, VOXELAN: a phantom study

**DOI:** 10.1093/jrr/rrac015

**Published:** 2022-04-25

**Authors:** Masahide Saito, Koji Ueda, Hidekazu Suzuki, Takafumi Komiyama, Kan Marino, Shinichi Aoki, Naoki Sano, Hiroshi Onishi

**Affiliations:** Department of Radiology, University of Yamanashi, Yamanashi 409-3898, Japan; Department of Radiology, University of Yamanashi, Yamanashi 409-3898, Japan; Department of Radiology, University of Yamanashi, Yamanashi 409-3898, Japan; Department of Radiology, University of Yamanashi, Yamanashi 409-3898, Japan; Department of Radiology, University of Yamanashi, Yamanashi 409-3898, Japan; Department of Radiology, University of Yamanashi, Yamanashi 409-3898, Japan; Department of Radiology, University of Yamanashi, Yamanashi 409-3898, Japan; Department of Radiology, University of Yamanashi, Yamanashi 409-3898, Japan

**Keywords:** image-guided radiotherapy (IGRT), optical imaging system, surface-guided radiotherapy (SGRT), breast cancer

## Abstract

The purpose of this study is to evaluate the detection accuracy of a 3-dimensional (3D) body scanner, VOXELAN, in surface-guided radiotherapy (SGRT) of each part of the human body using a whole-body human phantom. We used A Resusci Anne was used as the whole-body phantom. The detection accuracy of VOXELAN in a radiotherapy treatment room with a linear accelerator (LINAC) was evaluated for two reference images: reconstruction of the planning computed tomography (CT) image (CT reference) and scanning by VOXELAN before the treatment (scan reference). The accuracy of the translational and rotational directions was verified for four treatment sites (open face shell, breast, abdomen, and arm), using the magnitude of the 6D robotic couch movement as the true value. Our results showed that the detection accuracy improved as the displacement from the reference position decreased for all the sites. Using the scan reference, the average accuracy of the translational and rotational axes was within 1.44 mm and 0.41°, respectively, for all sites except the arms. Similarly, using the CT reference, the average accuracy was within 2.45 mm and 1.35°, respectively. Additionally, it was difficult for both reference images to recognize misalignment of the arms. In conclusion we discovered that VOXELAN achieved a high detection accuracy for the head with an open face shell, chest, and abdomen, indicating that the system is useful in a clinical setting. However, it is necessary to pay attention to location matching for areas with few features, such as surface irregularities and potential errors, when the reference image is created from CT.

## INTRODUCTION

Currently, image-guided radiotherapy (IGRT) is one of the most important techniques in radiotherapy. In recent years, surface-guided radiotherapy (SGRT) using surface scan and matching technology has been introduced, and its usefulness has been reported, especially in breast treatment [1]. Freislederer *et al.* mentioned that although many patients have the discomfort and emotional burden of tattoos, the use of this minimally invasive technique, a complete replacement of the patient’s tattoo with a markerless SGRT-based workflow could be a real prospect in the near future [2]. Furthermore, the technique is available for setting up the patient before treatment, and monitoring of the patient’s location during treatment. In stereotactic radiosurgery (SRS), it was reported that surface-guided SRS for benign skull base tumors produces clinical outcomes comparable to conventional frame-based SRS techniques while enhancing patient comfort [3]. Thus, SGRT use will continue to grow in the future due to its minimally invasive nature and versatility.

There are some commercially available devices for SGRT, such as AlignRT® (VisionRT, London, Great Britain), Sentinel/Catalyst™ (C-Rad, Uppsala, Sweden), IDENTIFY (Varian Medical Systems, Palo Alto, USA), and BrainLab ExacTrac Dynamic SGRT system (Brainlab AG, Germany) [2]. Moreover, a 3D body surface scanner, VOXELAN HEV-600M/RMS (Electronics Research & Development Corporation, Okayama, Japan), has begun to be used in radiotherapy in Japan, and it has been installed in our institute. While there are many reports on the accuracy and experience of using AlignRT® and Catalyst™ [3–7], there are no reports on the accuracy of VOXELAN for SGRT. Therefore, this study aimed to evaluate the detection accuracy of VOXELAN in each part of the human body using a whole-body human phantom.

## MATERIALS AND METHODS

### 3D body surface scanner

**Fig. 1:**
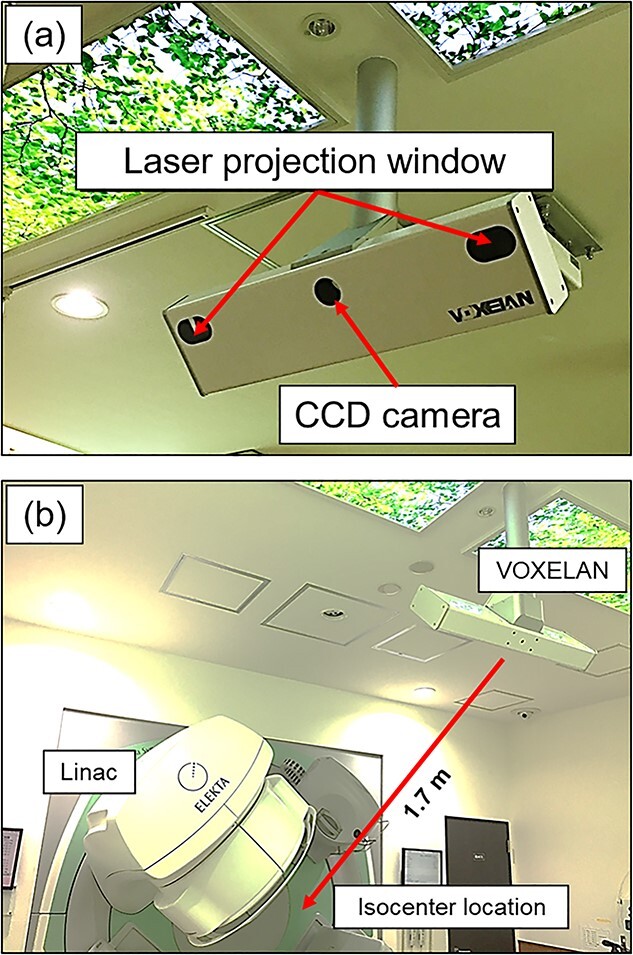
Overview of the VOXELAN system. (a) Overview of the camera, which consists of a CCD camera and two Laser projection windows. (b) Distance from LINAC, which is 1700 mm away from the isocenter location.

VOXELAN-HEV600M is the 3D body surface scanner system for SGRT use. Figure 1 shows an overview of the system installed at our institute. Worthy of note, the Elekta Synergy and on-rail computed tomography (CT) system had been installed in the treatment room in our institute. The main system consisted of one CCD camera and two laser projections, and was suspended from the room’s ceiling at 1700 mm from the isocenter. The CCD camera captures the laser projection by lateral scanning. The field of view is 600 mm × 450 mm × 600 mm, and measurement is performed in 0.5 sec/scan (including laser scanning and data output) with the resolution of 1280 × 1024 pixels. A user operates a PC and looks at a monitor in both the treatment and operation rooms. It was noted that the device’s position and angle will be determined by the vendor based on the facility’s environment, and the user cannot change this after installation.

**Fig. 2:**
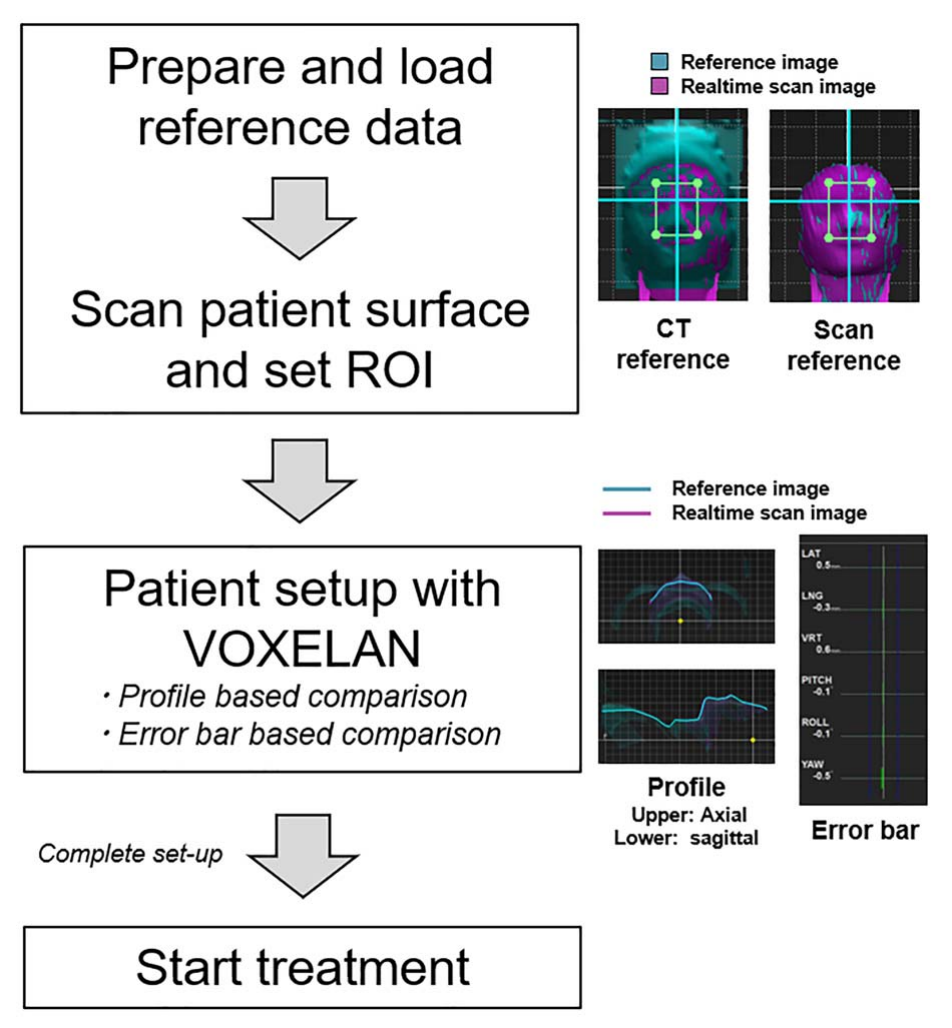
Workflow for using VOXELAN. The first step is preparation and loading of data. There are two ways to create a reference image, either from CT or by using the scanned image as the reference image. Then, patient setup with VOXELAN is performed by profile-based or error bar-based comparisons.

Figure 2 shows the workflow for using VOXELAN. The first step is the preparation and loading of data. There are two ways to create a reference image, either from CT (CT reference) or by using the scanned image (Scan reference) as the reference image. When using the CT reference, filtering and other clipping processes are performed in the VOXELAN software. It was possible to extract the appropriate body surface from the transferred treatment plan CT by adjusting the CT value and Window value for each patient, and it was also possible to remove the couch that is unnecessary for performing SGRT. The resolution of the output reference image from the software was 0.75 mm for both the CT reference and the scan reference.

Then, patient setup with VOXELAN was performed using profile-based or error bar-based comparisons. The profile-based comparison was performed by visual evaluation of the patient’s body surface, and the error bar-based comparisons were performed by displacement of each dimension between the reference and actual surface. For the detection of each region, only the displacement within the region of interest (ROI) was taken into account, and distant body parts outside the ROI were excluded from the calculation. Since the inclusion of invisible areas may affect the accuracy of the calculation, the ROI is generally defined as narrowly as possible to the focusing area. Furthermore, this ROI is a rectangle and can be changed to any size. It is noted that the ROI cannot be shaped into anything other than a rectangle, and no region within the ROI can be excluded from the analysis. The amount of displacement in each of the six directions was provided to the user in numerical or graphical form (bar). The amount of displacement was calculated by irradiating a laser beam onto the surface of the patient and measuring the 3D shape using a CCD camera. 3D shape measurement was calculated based on the principle of triangulation, using images acquired during the laser beam scanning as a series of images.

### Whole-body human phantom

**Fig. 3:**
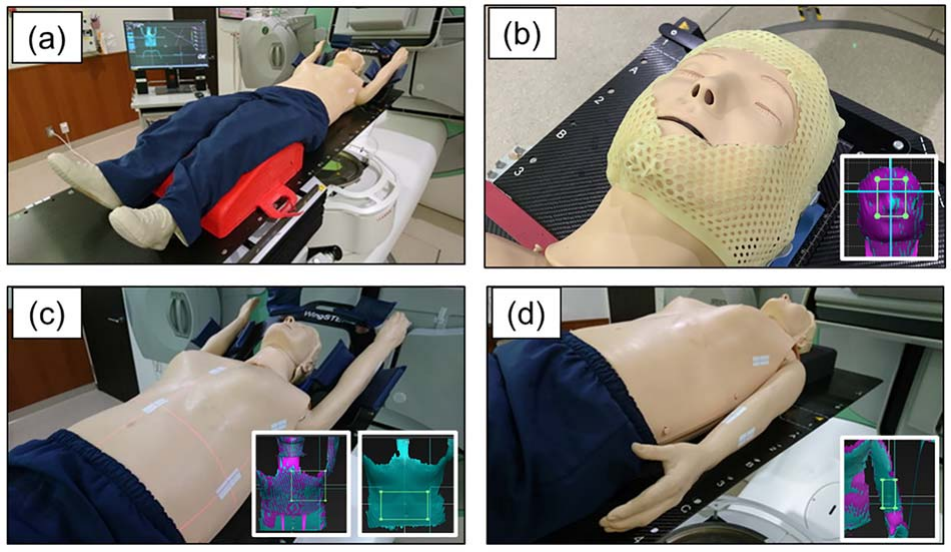
Scanned sites in this study. (a) Overview of the whole-body phantom, (b) set-up for the head with an open face shell, (c) set-up for the breast and abdomen, and (d) set-up for the arm.

A Resusci Anne (Laerdal Medical, Stavanger, Norway) was used as a whole-body human phantom. This phantom is usually used in simulating emergency medical services. To evaluate the detection accuracy at each body part, four sites (open face shell, breast, abdomen, and arm) were evaluated. The phantom and each evaluated site are shown in Fig. 3. For the evaluation of the head with an open face shell shown in Fig. 3(b), it was performed using a thermoplastic shell that removed the facial area and fixed only the chin and forehead. The breast and abdomen assessments shown in Fig. 3(c) were performed with both arms elevated. Wing Step (IT-V, Innsbruck, Austria) was used as the immobilization device. For the arms shown in (c), both arms were lowered to the side of the body.

### Evaluation of the set-up accuracy

The detection accuracy was evaluated for the four aforementioned sites using the following strategy. First, the phantom sets on the Hexapod® (Medical Intelligence) robotic couch, which is a computer-controlled couch top that allows for fine adjustments of patient position in each translational (longitudinal, lateral, vertical) and rotational (pitch, roll, yaw) axis, thus providing full six degree-of-freedom (6-DOF) positional corrections [8]. In this study, we used them to adjust the isocenter position. It should be noted that the tolerance of the indicated values of the Hexapod® was ±0.04 mm in the translational axis and ± 0.2° in the rotation axis, and these values were included as uncertainties in this study.

Then, by moving the couch, the phantom was displaced from −50 mm to +50 mm at 5 mm intervals for the translational axis and from −2.5° to 2.5° at 0.5° intervals for the rotational axis. Although VOXELAN can acquire scan data at about 0.5 sec/frame for positioning mode, in this study, an average of 20 frames was used as a stable value due to reducing data variation. These scan data were compared with the true couch movement values. In this study, Scan reference and CT reference were used as reference surface images; CT images were taken using an Aquilion LB (Canon Medical Systems, Otawara, Japan).

Moreover, we also conducted a verification under the combined situation of all axis movements in accordance with clinical practice. We performed the following three scenarios for the amount of movement: Scenario#1: all translational axis + 4 mm, all rotational axis + 2.5 deg. Scenario#2: all translational axis + 3 mm, all rotational axis + 2.0 deg. Scenario#3: all translational axis + 2 mm, all rotational axis + 1.5 deg.

## RESULTS

**Fig. 4:**
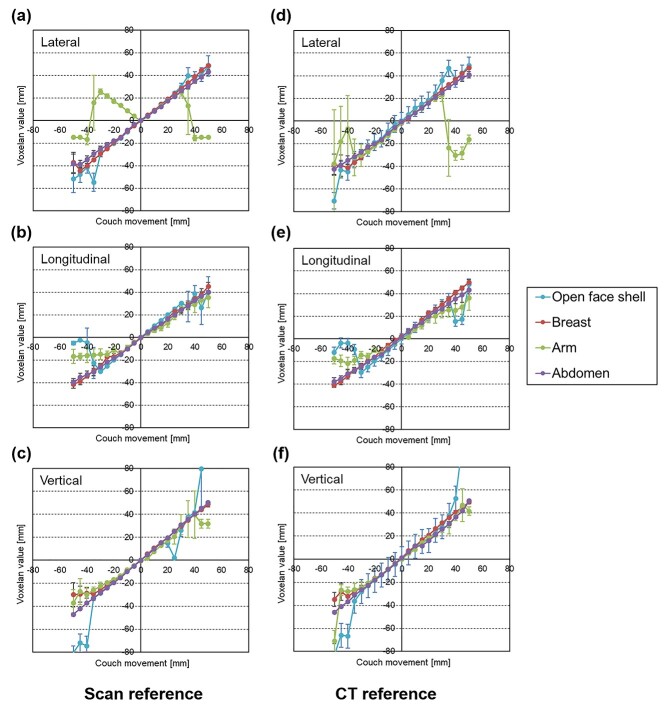
The detection accuracy of VOXELAN for the translational axis. The graph shows, for each site, the indicated value of VOXELAN on the vertical axis and the amount of bed movement on the horizontal axis. The left column shows the Scan reference, and the right column shows the CT reference. (a) and (d) show the Lateral accuracy, (b) and (d) show the Longitudinal accuracy, and (c) and (f) show the Vertical accuracy.

**Fig. 5:**
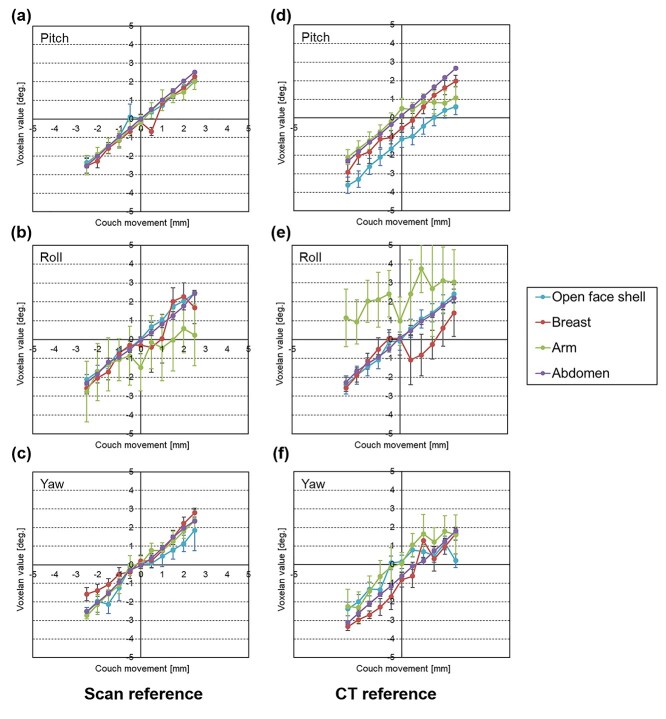
The detection accuracy of VOXELAN for the rotational axis. The graph shows, for each site, the indicated value of VOXELAN on the vertical axis and the amount of bed movement on the horizontal axis. The left column shows the Scan reference, and the right column shows the CT reference. (a) and (d) show the accuracy of Pitch, (b) and (d) show the accuracy of Roll, and (c) and (f) show the accuracy of Yaw.

Figure 4 shows the results of the detection accuracy of VOXELAN for the translational axis. The absolute average value and standard deviation of each value for 20 frames are shown. The detection accuracy tended to improve as the displacement from the reference position became smaller for all sites, regardless of whether the CT or VOXELAN reference image was used. However, as the displacement from the reference position became larger, the detection accuracy tended to decrease. This effect was particularly pronounced for the arm detection. In the same manner, Fig 5 shows the results for the rotational axis, showing that there was no correlation between the magnitude of the angle and the detection accuracy in all regions when either the CT or VOXELAN reference image was used.

**Fig. 6:**
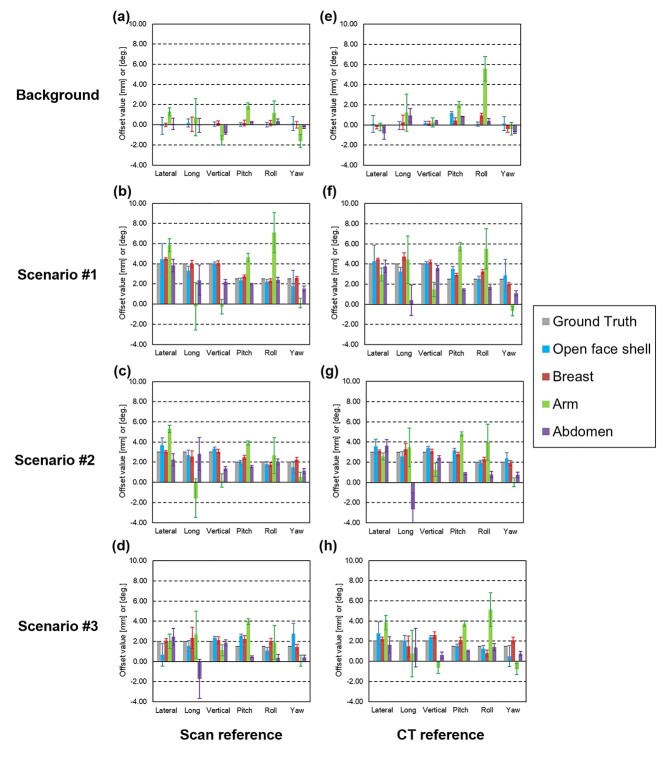
The detection accuracy for the combination of all directional movement. The graph shows the indicated value of VOXELAN on the vertical axis for each site. The left column shows the Scan reference, and the right column shows the CT reference. (a) and (e) show the accuracy of the background situation, (b) and (f) show scenario #1 (all translational axis + 4.0 mm, all rotational axis + 2.5°), (c) and (g) show scenario #2 (all translational axis + 3.0 mm, all rotational axis + 2.0°), and (d) and (h) show scenario #3 (all translational axis + 2.0 mm, all rotational axis + 1.5°).

As an additional study, we focused on the moving range of ±10 mm. Table 1 shows the results of the accuracy of each axis for the Scan reference and CT reference. For the Scan reference, the accuracy of the translational and rotational axes was within 1.44 mm and 0.41°, respectively, on average, for all sites except the arm. Conversely, the accuracy of the translational and rotational axes of the arm was within 6.22 mm and 0.90° on average, respectively.

For the CT reference, the accuracy of the translational and rotational axes was within 2.45 mm and 1.35°, respectively, on average, except for the arm. Conversely, for the arm, the accuracy of the translational and rotational axes was within 2.14 mm and 2.24° on average, respectively.

Figure 6 shows the results of the detection accuracy for the combination of all directional movements. The background is the result of the appropriate positioning of the phantom based on the CT reference; furthermore, the reference image for the Scan reference was also acquired under these conditions. The displacement of the translational and rotational axes in each scenario indicates the relative displacement from the background position. In all scenarios, the recognition error was within 1 mm/1° in most cases; however, large errors were observed in some of the translational axes of the Abdomen and each direction of the arm.

**Table 1:** The accuracy of each axis for the scan reference and CT reference on the moving range of ±10 mm

	SGRT with scan reference					SGRT with CT reference			
	Open face shell	Breast	Arm	Abdomen		Open face shell	Breast	Arm	Abdomen
*[Translational axis] detection accuracy within the movement of ± 10 mm range*									
Lateral [mm]	0.61 ± 0.65	0.41 ± 0.23	6.22 ± 6.89	1.44 ± 0.55		1.04 ± 0.77	2.20 ± 0.43	2.14 ± 0.59	1.34 ± 0.85
Long [mm]	0.21 ± 0.06	0.80 ± 0.69	1.35 ± 1.22	0.56 ± 0.68		0.18 ± 0.17	2.45 ± 1.59	1.81 ± 1.00	1.41 ± 0.37
Vertical [mm]	0.31 ± 0.25	0.46 ± 0.18	1.67 ± 1.14	0.12 ± 0.08		0.63 ± 0.41	1.47 ± 0.42	0.92 ± 0.67	1.09 ± 0.19
*[Rotational axis] Detection accuracy within the movement of ± 2.5 deg. range*									
Pitch [deg.]	0.21 ± 0.18	0.27 ± 0.30	0.16 ± 0.18	0.02 ± 0.02		1.35 ± 0.24	0.38 ± 0.17	0.51 ± 0.41	0.16 ± 0.02
Roll [deg.]	0.16 ± 0.14	0.41 ± 0.32	0.90 ± 0.71	0.14 ± 0.09		0.12 ± 0.08	0.84 ± 0.67	2.24 ± 1.07	0.15 ± 0.10
Yaw [deg.]	0.41 ± 0.27	0.35 ± 0.24	0.19 ± 0.11	0.07 ± 0.06		0.56 ± 0.62	0.98 ± 0.27	0.37 ± 0.24	0.66 ± 0.07

**Table 2:** The comparison with other devices (AlignRT and Catalyst) regarding accuracy

Devise	Author	Site	Reference-image	Ground-truth	Reported Accuracy (Absolute value, Maximum value among all axes)		Notes
					Translations (mm)	Rotations (°)	
AlignRT	Bert *et al.* [10]	Torso phantom	CT image	Couch	0.95	0.1	mean value
Catalyst	Walter *et al.* [11]	Thorax patient	CT image	CBCT	5.00	-	mean value
		Abdomen patient	CT image	CBCT	2.60	-	mean value
	Carl *et al.* [12]	Thorax patient	Scan image	CBCT	1.14	-	mean value
		Abdomen patient	Scan image	CBCT	1.99	-	Mean value
VOXELAN	This study	Breast phantom	Scan image /CT image	Couch	0.80 / 2.20	0.41 / 0.98	mean value
		Abdomen phantom	Scan image /CT image	Couch	1.44 / 1.41	0.14 / 0.66	mean value

## DISCUSSION

In the AAPM report of Task Group 147, two optical systems were mentioned [9]. AlignRT is one of the conventional methods of SGRT. In this system, a pseudo-random speck pattern is projected onto the patient’s body surface, and stereo cameras from three directions recognize the same points and calculate the coordinates of each point by triangulation processing. Catalyst is another system that has been in use conventionally. This system also provides optical image guidance with no radiation exposure by scanning the body surface in real time with cameras from three directions. This system can also display error-based projection mapping on the patient’s body surface during positioning, allowing for non-rigid positioning with visual guidance. These devices are used worldwide and can be linked to the couch attached to the conventional LINAC. Furthermore, most recently, the AAPM report of Task Group 302 was published [10]. As a commercial system, this report includes updates on the two devices above, as well as details on the new device, IDENTIFY (VARIAN). However, the VOXELAN used in this study is currently available only in Japan. The disadvantage is that the camera system of the cave crane is currently stand-alone and cannot be installed in multiple locations; hence, the couch must be moved manually. Conversely, it can be intuitively aligned using a profile display with a cross-sectional view of the patient and can be attached to any vendor’s treatment machine. Since there has been no previous report showing the basic performance of VOXELAN, in this study, we evaluated its performance using a whole-body phantom when used with Elekta Synergy.

In this study, we examined four sites, among which the arm was the most challenging to examine. There have been a few reports on extremities in the past [11]; however, the number of reports is limited and their use has not become widespread in clinical scenarios. In the case of the extremities, the range of detectable areas by SGRT is narrow, and there are few characteristics, suggesting that its use may be difficult. On the other hand, if there are easy-to-understand lesion-derived features on the surface, the positional accuracy may be improved compared to the results of this study.

As for the translational axis, the lateral and longitudinal directions tended to be slightly larger than the vertical direction in general. The reason for this may be the setting condition of the ROI. Since the ROI is set in the coronal plane, the position and size of the ROI are likely to affect the calculation accuracy for the two directions. Furthermore, it is not possible to rotate the ROI in the current VOXELAN system; thus, users will need to adjust the rectangular ROI to fit the shape of each site as much as possible. Therefore, the results of this study may also vary depending on the location and setting of the ROI.

Table 2 summarizes the comparison with other devices regarding accuracy. It can be seen that the accuracy of this study is comparable to other products [12–14]. Furthermore, the positioning accuracy tends to be worse when CT is used as a reference. When using the CT reference, it was possible to perform positioning within 2.2 mm accuracy for the head, chest, and abdomen; however, the overall accuracy was worse than when using the Scan reference. The accuracy of the CT reference creation depends on the resolution of the original CT image and the software’s body surface reconstruction algorithm. Therefore, as identified in this study, a 1–2 mm potential error may occur. Although more accurate software is required, the results of this study suggest that CT reference should be used in conjunction with other IGRT such as CBCT, and the use of Scan reference is recommended when more accurate position matching is necessary.

It is also noted that VOXELAN is a single camera system, and there are situations where blind spots are more likely to occur than existing systems that use cameras in three directions. Basically, for the isocenter position, within the FOV of 600 mm × 450 mm × 600 mm, visibility can be confirmed regardless of the angle of the gantry or couch, using VOXELAN. However, the edge of the FOV and the shadowed area may occur depending on the case, especially in the upstream direction of the gantry (i.e. gun direction). Moreover, this is the case for patients with a standard body shape, and the degree of the blind spot may vary depending on the patient’s body shape, such as obesity. Furthermore, it is susceptible to shadows caused by the gantry angle, especially around 0°. This study was conducted with the gantry angle fixed at 180° and the couch angle at 0°. However, since it was not possible to conduct experiments by changing these conditions, it will serve as a limitation of this study. In a clinical situation, we think that the effect of the rotation of the couch on the accuracy of VOXELAN needs to be confirmed for each case.

This study had some limitations. First of all, since this study was conducted on a phantom, the accuracy of each part on a real patient needs to be further verified. Next, the verification of the head position accuracy was done with the normal thermoplastic shell; however, it is also necessary to conduct a study with an open shell for body surface position matching. It is also worth considering patients for whom shells are unavailable, and a previous report using Catalyst reported successful treatment of whole-brain irradiation without shells, using a threshold of 3 mm/3° [15]. Moreover, this study was conducted in the treatment room of our hospital; thus, it should be noted that the accuracy may vary depending on the position and angle of the VOXELAN and the interference caused by the treatment device when used in other facilities.

## CONCLUSION

In this study, the positioning accuracy of the SGRT device, VOXELAN, was examined using a whole-body phantom. VOXELAN achieved a high positioning accuracy for the head with an open face shell, chest, and abdomen, indicating that the system is suitable for clinical use. However, it is necessary to pay attention to location matching for areas with few features such as, surface irregularities and potential errors when the reference image is created from CT.

## CONFLICT OF INTEREST

We collaborated with Hamano engineering and received funding and equipment support. The authors declare that they have no competing interests.

## FUNDING

Joint research funding from Hamano Engineering.

## References

[1] Betgen A, Alderliesten T, Sonke JJ et al. Assessment of set-up variability during deep inspiration breath hold radiotherapy for breast cancer patients by 3D-surface imaging. Radiother Oncol 2013;106:225–30. 23414819 10.1016/j.radonc.2012.12.016

[2] Freislederer P, Kugele M, Ollers M et al. Recent advanced in surface guided radiation therapy. Radiat Oncol 2020;15:187. 32736570 10.1186/s13014-020-01629-w PMC7393906

[3] Lau SK, Patel K, Kim T et al. Clinical efficacy and safety of surface imaging guided radiosurgery (SIG-RS) in the treatment of benign skull base tumors. J Neuro-Oncol 2017;132:307–12. 10.1007/s11060-017-2370-7 28120301

[4] Laaksomaa M, Sarudis S, Rossi M et al. AlignRT((R)) and catalyst in whole-breast radiotherapy with DIBH: is IGRT still needed? J Appl Clin Med Phys 2019;20:97–104. 10.1002/acm2.12553 PMC6414178 30861276

[5] Schonecker S, Walter F, Freislederer P et al. Treatment planning and evaluation of gated radiotherapy in left-sided breast cancer patients using the catalyst(TM)/sentinel(TM) system for deep inspiration breath-hold (DIBH). Radiat Oncol 2016;11:143. 27784326 10.1186/s13014-016-0716-5 PMC5080745

[6] Freislederer P, Reiner M, Hoischen W et al. Characteristics of gated treatment using an optical surface imaging and gating system on an Elekta LINAC. Radiat Oncol 2015;10:68. 25881018 10.1186/s13014-015-0376-x PMC4387684

[7] Kugele M, Edvardsson A, Berg L et al. Dosimetric effects of intrafractional isocenter variation during deep inspiration breath-hold for breast cancer patients using surface-guided radiotherapy. J Appl Clin Med Phys 2018;19:25–38. 10.1002/acm2.12214 PMC5768000 29139223

[8] Meyer J, Wilbert J, Baier K et al. Positioning accuracy of cone-beam computed tomography in combination with a HexaPOD robot treatment table. Int J Radiat Oncol Biol Phys 2007;67:1220–8. 17336222 10.1016/j.ijrobp.2006.11.010

[9] Willoughby T, Lehmann J, Bencomo JA et al. Quality assurance for nonradiographic radiotherapy localization and positioning systems: report of task group 147. Med Phys 2012;39:1728–47. 22482598 10.1118/1.3681967

[10] Al-Hallaq HA, Cerviño L, Gutierrez AN et al. AAPM task group report 302: surface guided radiotherapy. Med Phys 2022;49:e82–112. 10.1002/mp.15532 PMC9314008 35179229

[11] Gierga DP, Turcotte JC, Tong LW et al. Analysis of setup uncertainties for extremity sarcoma patients using surface imaging. Pract Radiat Oncol 2014;4:261–6. 25012835 10.1016/j.prro.2013.09.001

[12] Bert C, Metheany KG, Doppke K et al. A phantom evaluation of a stereo-vision surface imaging system for radiotherapy patient setup. Med Phys 2005;32:2753–62. 16266088 10.1118/1.1984263

[13] Walter F, Freislederer P, Belka C et al. Evaluation of daily patient positioning for radiotherapy with a commercial 3D surface-imaging system (catalyst). Radiat Oncol 2016;11:154. 27881158 10.1186/s13014-016-0728-1 PMC5122202

[14] Carl G, Reitz D, Schonecker S et al. Optical surface scanning for patient positioning in radiation therapy: a prospective analysis of 1902 fractions. Technol Cancer Res Treat 2018;17:1533033818806002. 30453842 10.1177/1533033818806002 PMC6243634

[15] Dekker J, Rozema T, Boing-Messing F et al. Whole-brain radiation therapy without a thermoplastic mask. Phys Imaging Radiat Oncol 2019;11:27–9. 33458273 10.1016/j.phro.2019.07.004 PMC7807553

